# P-1363. Cefiderocol Activity against Clinical Enterobacterales Isolates Carrying Metallo-β-lactamase Genes in United States and European Hospitals (2020–2023)

**DOI:** 10.1093/ofid/ofae631.1540

**Published:** 2025-01-29

**Authors:** Rodrigo E Mendes, Hank Kimbrough, Danielle Beekman, Cory Hubler, Joshua Maher, Helio S Sader, Mariana Castanheira

**Affiliations:** JMI Laboratories, North Liberty, Iowa; Element Materials Technology/Jones Microbiology Institute, North Liberty, Iowa; Element Materials Technology/Jones Microbiology Institute, North Liberty, Iowa; Element Materials Technology/Jones Microbiology Institute, North Liberty, Iowa; Element Materials Technology/Jones Microbiology Institute, North Liberty, Iowa; JMI Laboratories, North Liberty, Iowa; JMI Laboratories, North Liberty, Iowa

## Abstract

**Background:**

Cefiderocol (FDC) is a siderophore cephalosporin that uses the iron transport systems of Gram-negative bacteria to optimize cell entry. FDC is stable to hydrolysis by serine (e.g. ESBLs, KPC, and OXA-type carbapenemases) and metallo-β-lactamases (MBL). FDC and comparator activities were analyzed against Enterobacterales (ENT) carrying MBL genes, as part of the SENTRY Antimicrobial Surveillance Program.

**Figure:**
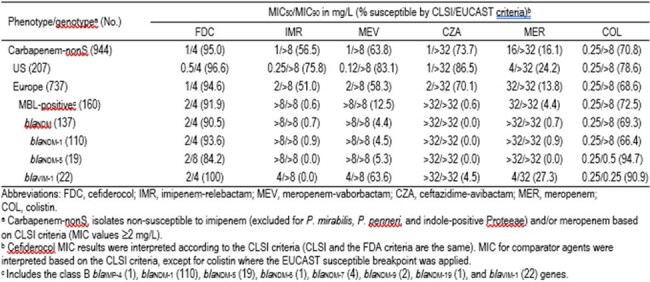

**Methods:**

32,052 ENT were collected from 35 sites in the US and 42 sites in the European (EU) regions during 2020–2023. Susceptibility (S) testing used broth microdilution with cation-adjusted Mueller-Hinton broth (CAMHB) for comparators and iron-depleted CAMHB for FDC. CLSI (same as the FDA) breakpoints were used for FDC; whereas CLSI were applied for comparators, except for colistin (EUCAST). ENT with MIC ≥2 mg/L (nonS based on CLSI criteria) for imipenem or meropenem were screened for β-lactamase genes.

**Results:**

Carbapenem-nonS isolates comprised 2.9% (944/32,052) of the population, of which 1.4% (207/15,147) and 4.4% (737/16,905) originated from US and EU sites, respectively. FDC (94.6–96.6%S) had equivalent MIC_90_ of 4 mg/L against all carbapenem-nonS isolates, and the US and EU subsets, whereas other agents had S ≤86.5% (Table). FDC had MIC_50/90_ of 2/4 mg/L against isolates carrying MBL genes, including the *bla*_NDM_ and *bla*_VIM-1_ subsets; in contrast, various other agents had off-scale MIC_90_ values (i.e. >8 mg/L). FDC MIC_90_ of 8 mg/L were obtained against isolates carrying *bla*_NDM-5_.

**Conclusion:**

FDC showed potent activity against carbapenem-nonS ENT and those subsets of isolates carrying MBL genes. This potent FDC activity is presented against a particular subset of resistant ENT, for which antibiotic treatment options are limited. FDC should be considered as an important option for the treatment of infections caused by these resistant organisms.

**Disclosures:**

**Rodrigo E. Mendes, PhD**, GSK: Grant/Research Support

